# Effects of localisation of uterine adenomyosis on outcome of *in vitro* fertilisation/intracytoplasmic sperm injection fresh and frozen-thawed embryo transfer cycles: a multicentre retrospective cohort study

**DOI:** 10.1186/s12958-021-00764-7

**Published:** 2021-06-04

**Authors:** Takuya Iwasawa, Toshifumi Takahashi, Eri Maeda, Koichi Ishiyama, Satoshi Takahashi, Ryota Suganuma, Koki Matsuo, Masahito Tachibana, Rie Fukuhara, Hiromitsu Shirasawa, Wataru Sato, Yukiyo Kumazawa, Yukihiro Terada

**Affiliations:** 1 Department of Obstetrics and Gynecology, Akita University Graduate School of Medicine, 010-8543 Akita, Japan; 2 Department of Obstetrics and Gynecology, Omagari Kousei Medical Center, 014- 0027 Daisen, Akita Japan; 3 Fukushima Medical Center for Children and Women, Fukushima Medical University, 960-1295 Fukushima, Japan; 4 Department of Environmental Health Science and Public Health, Akita University Graduate School of Medicine, 010-8543 Akita, Japan; 5 Department of Radiology, Akita University Graduate School of Medicine, 010-8543 Akita, Japan; 6 Department of Radiology, Hiraka General Hospital, 013-8610 Akita, Japan; 7 Department of Obstetrics and Gynecology, Fukushima Medical University School of Medicine, 960-1295 Fukushima, Japan; 8 Department of Obstetrics and Gynecology, Yamagata University Faculty of Medicine, 990-9585 Yamagata, Japan; 9 Department of Obstetrics and Gynecology, Tohoku University School of Medicine, 980-8574 Sendai, Miyagi Japan; 10 Department of Obstetrics and Gynecology, Hirosaki University Graduate School of Medicine, 036-8563 Hirosaki, Aomori Japan

**Keywords:** Localisation, Adenomyotic lesion, Adenomyosis, Magnetic resonance imaging, *in vitro* fertilisation/intracytoplasmic sperm injection, Embryo transfer

## Abstract

**Background:**

Uterine adenomyosis is a benign disease, common among women in their 40 and 50 s, characterised by ectopic endometrial tissue in the uterine myometrial layer. Adenomyosis causes infertility and has a negative effect on the outcomes of *in vitro* fertilisation (IVF)/intracytoplasmic sperm injection (ICSI) embryo transfer (ET) cycles. It has also been reported to have different characteristics depending on the adenomyotic lesion localisation. The effect of its localisation on IVF/ICSI-ET outcomes is unclear. This study aimed to investigate whether adenomyotic lesion localisation, assessed using magnetic resonance imaging (MRI), was associated with outcomes of IVF/ICSI-ET cycles.

**Methods:**

This multicentre, joint, retrospective cohort study analysed the medical records of 67 infertile patients with adenomyosis who underwent IVF/ICSI with fresh and frozen-thawed ET at five participating facilities from January 2012 to December 2016 and for whom MRI data were available. Fifteen patients were excluded; therefore, the MRI data of 52 patients were evaluated by two radiologists. We assessed the localisation of and classified adenomyotic lesions into advanced (invades the full thickness of the uterine myometrium), extrinsic (localised on the serosal side), and intrinsic (localised on the endometrial side) subtypes.

**Results:**

There were 40 advanced, nine extrinsic, and three intrinsic cases, and the outcomes of 100, 27, and nine ET cycles, respectively, were analysed. Pregnancy loss/clinical pregnancy and live birth rates of the advanced, extrinsic, and intrinsic groups were 64 % (16/25) and 9 % (9/100), 33.3 % (3/9) and 22.2 % (6/27), and 50 % (1/2) and 11.1 % (1/9), respectively. A logistic regression analysis adjusted for age, prior miscarriage, and body mass index showed that the extrinsic group had fewer pregnancy losses (odds ratio 0.06; 95 % confidence interval [CI]: 0.00–0.54, p = 0.026) and more live births (odds ratio 6.05; 95 % CI: 1.41–29.65, p = 0.018) than the advanced group.

**Conclusions:**

Adenomyotic lesions exert different effects on IVF/ICSI-ET outcomes. Thus, MRI assessments of adenomyosis in infertile patients are beneficial. Establishment of treatment plans based on adenomyotic lesion localisation should be considered.

**Supplementary Information:**

The online version contains supplementary material available at 10.1186/s12958-021-00764-7.

## Background

Uterine adenomyosis is a benign disease that is common among women in their 40 and 50 s [1]. It is characterised by ectopic endometrial tissue in the uterine myometrial layer [2]. There are several theories on the origins of adenomyosis, such as direct invasion [3], tissue injury and repair [4], Müllerian remnants that develop into *de novo* ectopic endometrial tissue [3], endometrial stem/progenitor cells [5], and epithelial-to-mesenchymal transition [6, 7]. Therefore, adenomyosis may present different characteristics depending on the origin.

Adenomyosis becomes a clinical problem when the patient presents with symptoms, including hypermenorrhoea, dysmenorrhoea, and infertility [1, 8, 9]. A systematic review regarding the outcomes of *in vitro* fertilisation/intracytoplasmic sperm injection-embryo transfer (IVF/ICSI-ET) reported that patients with adenomyosis had a 28 % lower clinical pregnancy rate and a two-fold higher miscarriage rates than patients without adenomyosis [10]. However, the effects of adenomyosis on IVF/ICSI-ET outcomes remain controversial. This may be because of the general lack of consensus on the diagnostic criteria for adenomyosis; thus, various forms of adenomyosis are not classified by severity or localisation, and this condition is currently treated as a homogenous disease [10, 11].

A histological diagnosis of adenomyosis is definitive [2], yet a definitive diagnosis generally cannot be established for patients requiring uterus-preserving therapy. Hence, adenomyosis is generally diagnosed comprehensively based on symptoms and diagnostic ultrasonography and/or by magnetic resonance imaging (MRI). On MRI, the diagnostic criteria for adenomyosis include a maximal junctional zone (JZ) thickness ≥ 12 mm, maximal JZ thickness/myometrium thickness measured at the same region ≥ 40 %, and high-intensity myometrial foci < 3 mm within the myometrium [11, 12]. The diagnosis by MRI includes fewer criteria than the diagnosis by ultrasonography [11, 13]. Moreover, MRI is considered a better and more objective diagnostic tool for adenomyosis than ultrasonography [11].

An established classification system is required to predict the individual therapeutic outcomes and prognosis of adenomyosis [11, 14]. Using MRI, Kishi et al. [15] classified adenomyotic lesions into four subtypes according to their localisation; using this method, the authors found common characteristics and pathological features in patients with a similar classification [16]. However, there are few reports regarding IVF/ICSI-ET outcomes and how they vary among the four different lesion types. Studies have reported that the risks of miscarriage, preterm delivery, preeclampsia, and placental malposition in the second trimester increased in pregnant patients with adenomyosis [17, 18]; however, the effects of these subtypes on outcomes remain unknown. Thus, this study aimed to investigate whether the localisation of adenomyotic lesions, as assessed by two experienced radiologists using MRI, was associated with outcomes of IVF/ICSI-ET and to report the perinatal prognoses of the four clinical subtypes of adenomyotic lesions.

## Methods

### Design and patients

This multicentre, joint, retrospective cohort study analysed data obtained from the medical records of 67 infertile patients with adenomyosis from five facilities (Department of Obstetrics and Gynecology, Akita University Graduate School of Medicine; Department of Obstetrics and Gynecology, Hirosaki University Graduate School of Medicine; Department of Obstetrics and Gynecology, Tohoku University School of Medicine; Department of Obstetrics and Gynecology, Yamagata University Faculty of Medicine; and Department of Obstetrics and Gynecology Fukushima Medical University School of Medicine) between January 2012 and December 2016. These five facilities all belong to the Tohoku Unit of Reproductive Medicine. We analysed patients who were diagnosed with adenomyosis by ultrasonography, underwent IVF/ICSI fresh or frozen-thawed ET, and had available MRI data. The IVF/ICSI fresh and frozen-thawed ET protocol was determined by the participating gynaecologist at each facility.

This study was conducted with the approval of the ethics committees of the five participating facilities. The ethics committees waived the requirement for informed consent because the study reviewed anonymised data.

### Data collection

**Fig. 1:**
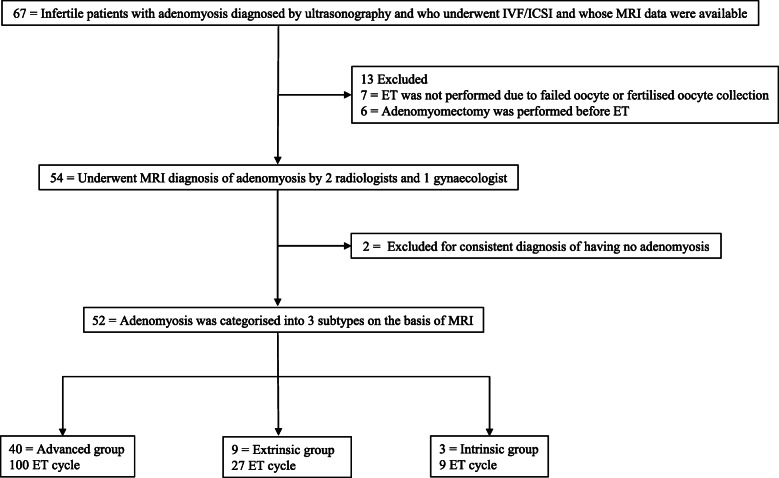
Flowchart of the participant selection and categorisation throughout the study. MRI, magnetic resonance imaging; ET, embryo transfer

We excluded patients whose oocytes could not be collected, whose viable fertilised oocytes or frozen embryos could not be collected for transfer, who had undergone conservative surgery for adenomyosis before the ET, and who were not diagnosed with adenomyosis by the two radiologists using the uniform MRI diagnostic criteria (Fig. 1). Infertility was diagnosed by a gynaecologist specialised in assisted reproductive technology (ART) at each facility. Good-quality embryos were defined as Veeck class G1-2 for cleavage-stage embryos [19], Tao class M3 or 4 for morulae [20], and Gardner class 3BB and higher for blastocysts [21]. Patients classified as having received pre-transfer gonadotropin-releasing hormone (GnRH) treatment were defined as those who underwent GnRH treatment for two or more cycles up to three months prior to ET. A final diagnosis and classification were established according to the diagnostic criteria of this study based on the MR images, which were taken at each facility separately by two experienced radiologists who were blinded to the patient characteristics and IVF/ICSI-ET outcomes. When the judgment of the two radiologists differed, a third-party gynaecologist, also blinded to the patient’s characteristics and IVF/ICSI-ET outcomes, made the final diagnosis and classification.

### MRI diagnosis

MRI findings were assessed for any ovarian endometriomas measuring ≥ 2 cm. Adenomyosis was diagnosed on MR images when one or more of the following diagnostic criteria were met [11, 12]: (1) *JZ thickness*, the JZ was measured on a midsagittal image through the long axis of the uterus on T2-weighted images; that is, the maximal JZ thickness was ≥ 12 mm or the maximal JZ thickness to myometrial thickness ratio was ≥ 40 %; (2) *microcysts*, round cystic foci varying from 2 to 7 mm in diameter were embedded within the ill-defined myometrial lesion on T1- or T2-weighted images. Ovarian endometrioma was diagnosed if a lesion with a T1 high-intensity region measuring ≥ 2 cm was present in the fat-suppressed MR images.

### Classification according to the localisation of adenomyotic lesions

**Fig. 2:**
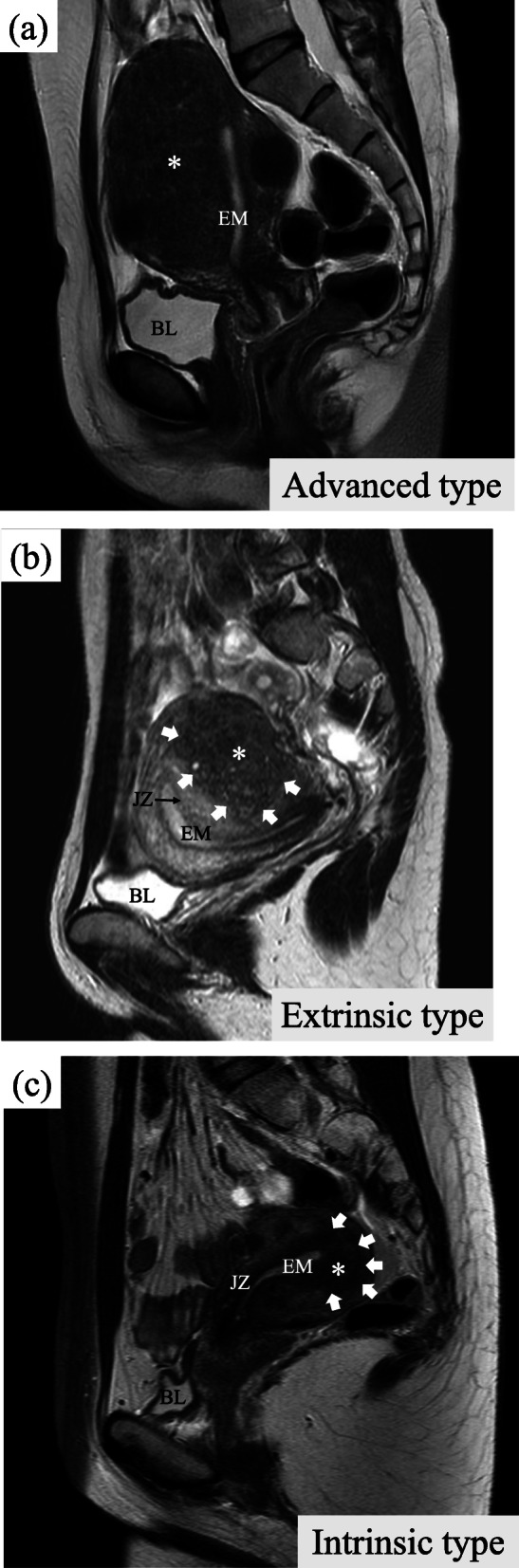
Representative sagittal T2-weighted MRI for each subtype. a Advanced type: the adenomyotic lesion invades the endometrium from the serosa through the full thickness of the myometrium. b Extrinsic type: the adenomyotic lesion is localised to the serosal side of the posterior wall of the uterus. A normal myometrium exists between the adenomyotic lesion and the junctional zone. The arrows show the border between the adenomyotic lesion and the uterine myometrium. c Intrinsic type: the adenomyotic lesion is localised to the endometrial side. The myometrium external to the lesion is intact. The arrows show the border between the adenomyotic lesion and uterine myometrium. MRI, magnetic resonance imaging; EM, endometrium; JZ, junctional zone; BL, bladder. The asterisks show the adenomyotic lesion

Classification by lesion localisation on MRI scans was performed according to the methods of Kishi et al. [15]. No patients were found to have an intramural type-resembling adenomyoma. Thus, the patients were classified into one of the following three types: (1) *advanced type*, the lesion invaded the full thickness of the uterine myometrium (Fig. 2a); (2) *extrinsic type*, the lesion was located on the external side of the uterine myometrium with parts without normal myometrium on the serosal side of the lesion and parts with normal myometrium on the endometrial side (Fig. 2b); and (3) *intrinsic type*, the lesion was located on the internal side of the uterine myometrium with parts without normal myometrium on the endometrial side of the lesion and with normal myometrium on the serosal side (Fig. 2c).

### Accuracy of diagnosis of adenomyosis by MRI

The concordance rate between the MRI-based diagnoses of adenomyosis by the two radiologists was 94.2 % (49/52). The kappa coefficient of inter-rater reliability was 0.55. There was an 89.8 % (44/49) diagnostic concordance rate of the adenomyotic lesion localisation between the two radiologists. The weighted kappa coefficient was 0.96.

### Study outcomes

The outcome measures were the clinical pregnancy rate per ET (cycle), clinical pregnancy loss rate per clinical pregnancy, and live birth rate per ET (cycle). The incidences of caesarean delivery, preterm labour, threatened preterm labour, cervical incompetence, placenta previa, placenta accreta, foetal growth restriction, and preeclampsia were evaluated as measures of perinatal prognosis. Clinical pregnancy was defined as the presence of at least one intrauterine gestational sac on the vaginal ultrasound examination during the eighth gestational week.

### Statistical analyses

All statistical analyses were performed using the R software package (version 4.0.0) (R Foundation for Statistical Computing, Vienna, Austria). Continuous variables were expressed as medians with ranges and were analysed using the non-parametric Kruskal-Wallis test. Categorical variables were analysed using the Fisher’s exact test. Logistic regression analysis was performed on clinical pregnancy, clinical pregnancy loss, and live birth rates to normalise the distribution of the results. A p-value < 0.05 was considered statistically significant.

## Results

A flow diagram of participant selection is shown in Fig. 1. Of the 67 patients, 15 were excluded for the following reasons: oocytes, fertilised oocytes, or frozen embryos could not be obtained (*n* = 7) and, thus, ET could not be performed during the study period; conservative surgery was performed for adenomyosis before ET (*n* = 6); and the diagnostic criteria of adenomyosis by MRI, as diagnosed by the two radiologists, were not met (*n* = 2) (Fig. 1).

The lesion localisations in each group are shown in Table 1. In this study, 40 (76.9 %), 9 (17.3 %), and 3 (5.8 %) patients were found to have advanced, extrinsic, and intrinsic lesion types, respectively.

**Table 1:** Classification of the adenomyotic lesions according to their localisations

	Advanced group (*n* = 40)	Extrinsic group (*n* = 9)	Intrinsic group (*n* = 3)
**Circumferential, n (%)**	8 (20)	1 (11.1)	0
**Abdominal side, n (%)**	4 (10)	1 (11.1)	1 (33.3)
**Dorsal side, n (%)**	28 (70)	7 (77.8)	2 (66.7)

In the advanced lesion group, the lesion was circumferential in eight, located on the abdominal side (anterior) in four, and located on the dorsal side (posterior) in 28 patients. In the extrinsic group, the lesion was circumferential in one, located on the abdominal side in one, and located on the dorsal side in seven patients. None of the patients in the intrinsic group had a circumferential lesion, whereas one had a lesion on the abdominal side, and two had lesions on the dorsal side.

The patient characteristics for all groups are shown in Table 2. No significant differences in age, duration of infertility, gravidity, parity, miscarriages, the number of previous IVF/ICSI-ET cycles prior to this study, previous uterine surgery (caesarean section and myomectomy), hypermenorrhoea, or dysmenorrhoea were observed among the three groups. The body mass index (BMI) was significantly lower in patients with extrinsic adenomyosis than in those with other types (*p* = 0.018). The most common cause of infertility was endometriosis in 60 and 44 % of the patients in the advanced and extrinsic groups, respectively. None of the patients with intrinsic adenomyosis experienced infertility caused by endometriosis. Moreover, no significant differences were observed among the three groups in terms of the causes of infertility. The proportion of patients in the advanced, extrinsic, and intrinsic groups with a history of surgery for ovarian endometrioma were 32.5 %, 77.8 %, and 33.3 %, respectively (p = 0.035). The rates of ovarian endometrioma assessed using MR images in the advanced, extrinsic, and intrinsic groups were 70 %, 100 %, and 0 %, respectively (*p* = 0.004).

**Table 2:** Patient characteristics

	Advanced group (*n* = 40)	Extrinsic group (*n* = 9)	Intrinsic group (*n* = 3)	*P-value*
**Age (years)**	36 (26–41)	37 (30–39)	35 (31–36)	0.403
**Body mass index (kg/m**^**2**^**)**	23 (18.1–32.5)	19.9 (18.1–23.3)	23.5 (19.5–25.8)	0.018
**Duration of infertility (months)**	41.5 (6–151)	42 (0–66)	45 (31–90)	0.519
**Cause of infertility, n (%)**				0.092
**Endometriosis**	24 (60)	4 (44.4)	0	
**Tubal**	2 (5)	1 (11.1)	0	
**Anovulation**	0	0	1 (33.3)	
**Mixed**	9 (22.5)	3 (33.3)	1 (33.3)	
**Unexplained**	5 (12.5)	1 (11.1)	1 (33.3)	
**Gravidity, n (%)**	17/40 (42.5)	4/9 (44.4)	2/3 (66.7)	0.879
**Parity, n (%)**	7/40 (0–1)	3/9 (0–1)	0	0.411
**Miscarriage, n (%)**	13/40 (32.5)	1/9 (11.1)	2/3 (66.7)	0.199
**Prior IVF/ICSI-ET treatment cycle**	0 (0–9)	0 (0–8)	0 (0–6)	0.933
**Prior uterine surgery, n (%)**	6/40 (15)	2/9 (22.2)	0	0.777
**Prior surgery for endometrioma, n (%)**	13/40 (32.5)	7/9 (77.8)	1/3 (33.3)	0.035
**Presence of endometrioma, n (%)**	28/40 (70)	9/9 (100)	0	0.004
**Hypermenorrhoea, n (%)**	28/40 (70)	5/9 (55.5)	1/3 (33.3)	0.324
**Dysmenorrhoea, n (%)**	35/40 (87.5)	7/9 (77.8)	2/3 (66.7)	0.354

*IVF/ICSI-ET* *in vitro* fertilisation/intracytoplasmic sperm injection-embryo transfer

Data related to IVF/ICSI-ET for the three groups are shown in Table 3. No significant differences were observed in oocyte retrieval cycles. The numbers of oocytes retrieved per collection for the advanced, extrinsic, and intrinsic groups were 2 (0–15), 4 (1–11), and 8 (8–14), respectively. No significant differences were observed among the groups regarding the use of IVF or ICSI for fertilisation. No significant differences in the number of transfer cycles during the study period were observed. The median (range) number of transferred embryos per cycle was 1 (1–2) for all groups. The proportions of fresh and frozen-thawed ET were not significantly different within each group. Furthermore, 100 % (n = 9) of the transferred embryos in the intrinsic group, 78 % in the advanced group, and 63 % in the extrinsic group were of good quality. GnRH was administered before ET in 20 patients (20 %) in the advanced group and in one patient (3.7 %) in the extrinsic group, but none in the intrinsic group.

**Table 3:** Characteristics of IVF/ICSI-ET

	Advanced group (*n* = 40)	Extrinsic group (*n* = 9)	Intrinsic group (*n* = 3)	*P-value*
**Number of oocyte retrieval cycles**	2 (1–9)	2 (1–4)	1	0.210
**Type of controlled ovarian stimulation, n (%)**				0.068
**Short**	49/111 (44.1)	4/21 (19)	0/3	
**Long**	26/111 (23.4)	11/21 (52.4)	1/3 (33.3)	
**Ultra-long**	16/111 (14.4)	2/21 (9.5)	1/3 (33.3)	
**GnRH antagonist**	15/111 (13.5)	3/21 (14.3)	1/3 (33.3)	
**Others**	5/111 (4.5)	1/21 (4.8)	0/3	
**Total dose of gonadotropin used (IU)**	2400 (225–6750)	2250 (600–2250)	1425 (1025–2625)	0.162
**Number of oocytes retrieved**	2 (0–15)	4 (1–11)	8 (8–14)	0.008
**Fertilisation method, n (%)**				0.616
**ICSI**	35/106 (33.0)	6/21 (28.6)	2/3 (66.7)	
**IVF**	67/106 (63.2)	14/21 (66.7)	1/3 (33.3)	
**Mixed**	4/106 (3.8)	1/21 (4.7)	0/3	
**Number of ET cycles**	2 (1–8)	3 (2–5)	3 (2–4)	0.129
**Number of ET/cycles**	1 (1–2)	1 (1–2)	1 (1–2)	0.802
**ET type, n (%)**				0.083
**Fresh**	49/100 (49)	11/27 (40.7)	1/9 (11.1)	
**Frozen-thawed**	51/100 (51)	16/27 (59.3)	8/9 (88.9)	
**Embryo quality, (%)**				0.034
**Good**	77/100 (77)	16/27 (59.3)	9/9 (100)	
**Long-term pituitary down-regulation before ET, n (%)**	20/100 (20)	1/27 (3.7)	0/9	0.048

*IVF* *in vitro* fertilisation; *ICSI* intracytoplasmic sperm injection; *ET* embryo transfer; *GnRH* gonadotropin-releasing hormone

The outcomes of IVF/ICSI fresh and frozen-thawed ET are shown in Table 4. The clinical pregnancy rates were 25 % (25/100), 33.3 % (9/27), and 22.2 % (2/9) for the advanced, extrinsic, and intrinsic groups, respectively. Their pregnancy loss rates were 64 % (16/25), 33.3 % (3/9), and 50 % (1/2), respectively, whereas their live birth rates were 9 % (9/100), 22.2 % (6/27), and 11.1 % (1/9), respectively.

**Table 4:** IVF/ICSI fresh and frozen-thawed ET outcomes

	Advanced group (*n* = 40)	Extrinsic group (*n* = 9)	Intrinsic group (*n* = 3)	P*-*value
**Clinical pregnancy rate/ET, n (%)**	25/100 (25)	9/27 (33.3)	2/9 (22.2)	0.701
**Pregnancy loss/clinical pregnancy, n (%)**	16/25 (64)	3/9 (33.3)	1/2 (50)	0.229
**Pregnancy loss after 12 weeks, n (%)**	4/16 (25 %)	0/3	0/1	n.s.
**Live birth rate/ET, n (%)**	9/100 (9)	6/27 (22.2)	1/9 (11.1)	0.137

*IVF/ICSI* *in vitro* fertilisation/intracytoplasmic sperm injection; *ET* embryo transfer; *n.s.* not significant

The logistic regression analysis results are shown in Table 5. The relationships between localisation of the adenomyotic lesion and clinical pregnancy, pregnancy loss, and live birth rates, which were adjusted for age, prior miscarriage, and BMI were investigated. After adjusting for these factors, no significant relationships were observed between the localisation of the adenomyotic lesion and clinical pregnancy rate. There were few patients with extrinsic- or intrinsic-type lesions; thus, these two groups could not be compared. Compared with the advanced group, the extrinsic group had a significantly lower rate of pregnancy loss with an odds ratio (OR) of 0.66 (95 % confidence interval [CI]: 0.00–0.54, p = 0.026). Furthermore, compared to the advanced group, the extrinsic group had a significantly higher rate of live birth with an OR of 6.05 (95 % CI: 1.41–29.65, p = 0.018.

**Table 5:** Logistic regression of IVF/ICSI fresh and frozen-thawed ET outcomes

	Types of adenomyosis	Crude odds ratio	95 % CI	P-value	Adjusted OR^a^	95 % CI	P-value
Clinical pregnancy	Advanced	1.00	-	-	1.00	-	-
	Extrinsic	1.50	0.58–3.71	0.387	1.35	0.47–3.77	0.564
	Intrinsic	1.50	0.30–6.14	0.586	1.31	0.25–5.65	0.725
Pregnancy loss	Advanced	1.00	-	-	1.00	-	-
	Extrinsic	0.281	0.05–1.34	0.122	0.06	0.00–0.54	0.026
	Intrinsic	0.563	0.02–15.38	0.696	1.83	0.06–57.62	0.701
Live birth	Advanced	1.00	-	-	1.00	-	-
	Extrinsic	2.89	0.89–8.93	0.067	6.05	1.41–29.65	0.018
	Intrinsic	1.26	0.06–8.12	0.834	0.83	0.04–5.64	0.871

^a^Adjusted odds ratio (OR) for age, prior miscarriage, and body mass index. *IVF/ICSI* *in vitro* fertilisation/intracytoplasmic sperm injection; *ET* embryo transfer; *CI* confidence interval

In contrast to the extrinsic and intrinsic groups, for which no pregnancy losses were recorded at 12 weeks or later, pregnancy loss was observed in 25 % (4/16) of the patients in the advanced group.

The perinatal prognoses of the 16 patients who had live births are shown in Table 6. Ten patients delivered by caesarean Sec. (62.5 %), preterm labour occurred in three patients (18.6 %), and threatened premature labour occurred in eight patients (50 %). Placenta previa, foetal growth restriction, and preeclampsia occurred in one patient with an advanced-type lesion. No significant differences were observed between these patients in terms of the localisation of the adenomyotic lesion.

**Table 6:** Perinatal prognoses

	Total	Advanced group	Extrinsic group	Intrinsic group
**Caesarean delivery, n (%)**	10/16 (62.5)	7/9 (77.8)	3/6 (50)	0/1
**Preterm labour, n (%)**	3/16 (18.8)	1/9 (11.1)	2/6 (33.3)	0/1
**Threatened preterm labour, n (%)**	8/16 (50)	5/9 (55.6)	3/6 (50)	0/1
**Cervical incompetency, n (%)**	2/16 (12.5)	1/9 (11.1)	1/6 (16.7)	0/1
**Placenta previa, n (%)**	1/16 (6.2)	1/9 (11.1)	0/6	0/1
**Foetal growth restriction, n (%)**	1/16 (6.2)	1/9 (11.1)	0/6	0/1
**Preeclampsia, n (%)**	1/16 (6.2)	1/9 (11.1)	0/6	0/1

## Discussion

In this retrospective, multicentre joint study, two double-blinded radiologists diagnosed adenomyosis based on MRI findings using the same diagnostic criteria and classified patients according to the localisation of the adenomyotic lesions. The IVF/ICSI fresh and frozen-thawed ET outcomes were compared according to these classifications. The extrinsic group had fewer pregnancy losses and more live births than the advanced group, whereas the advanced group had a greater proportion of patients with miscarriages at or after 12 weeks.

While some studies have reported negative effects for adenomyosis on IVF/ICSI-ET outcomes [22–26], others report no negative effects [27, 28]. These data suggest that additional research is needed to clarify the effects of adenomyosis on IVF/ICSI-ET outcomes.

One cause of the discrepancies in the data may be the differences between the methods used to diagnose adenomyosis; some studies used ultrasonography data [25–28], whereas others used MRI data [22, 23] or a mix of ultrasonography and MRI data [26, 29]. Furthermore, the uniform assessment of adenomyosis, despite variations in severity or localisation, may have contributed to the discrepancies in the data.

A definitive diagnosis of adenomyosis can only be established histologically. However, infertile patients with adenomyosis cannot undergo hysterectomy; thus, they should be diagnosed clinically based on their symptoms or using diagnostic imaging modalities.

According to a 2018 review, the pooled sensitivities and specificities of ultrasound diagnosis were 0.72–0.82 and 0.81–0.85, respectively, whereas those of MRI diagnosis were 0.77 and 0.89, respectively [11]. Thus, the diagnostic accuracy rates of ultrasound and MRI diagnoses for adenomyosis were almost equal [11, 30]. Performing ultrasonography for the diagnosis of adenomyosis is beneficial due to its lower cost and convenience during routine examinations. Conversely, it is disadvantageous due to its subjectivity and reliance on the capacity of the observer to diagnose. The high heterogeneity of the diagnostic criteria [11, 13] only makes ultrasonography more disadvantageous.

However, the benefits of using MRI to diagnose adenomyosis include its superior objectivity, as it provides other detailed intrapelvic information, which allows the concurrent diagnosis of ovarian endometrioma. The high degree of concordance in the classifications made by the two radiologists in our study, according to adenomyotic lesion localisations, can be attributed to the high objectivity of MRI. However, the diagnostic concordance rate was not 100 % (49/52). In one of three discordant patients the two radiologists had different judgments regarding the diagnosis of myoma. Patients with adenomyosis often develop coexisting myomas [2, 13]; in some patients, differentiating between a myoma and an adenomyotic lesion can be difficult as there is no concrete method for their differentiation using MRI data; nonetheless, MRI is reported to be superior to ultrasonography for the differential diagnosis [30]. Although ultrasonography is effective in screening for adenomyosis, MRI is more desirable for assessing adenomyosis because it generates more information and, therefore, increases objectivity.

Recent studies have sought to establish a concrete adenomyosis classification; in particular, they have proposed a classification system based on lesion localisation [11, 14]. The important elements of classification are to ensure that common characteristics are observed within all classifications and that the classifications are not subdivided excessively. The system reported by Kishi et al. [15] appears to meet such requirements because they classified adenomyosis into four types according to lesion localisation, and each type was characterised by specific traits.

With regard to the characteristics of patients with intrinsic-type lesions, pregnancy termination (curettage) rate was 32.3 %, higher than that of patients with other lesion types, suggesting that the invasion of endometrial tissues due to the rupture of a barrier at the endometrial-myometrial interface may be involved in the onset of adenomyosis [15]. Patients with the extrinsic type had a higher rate of lesions on the dorsal side (96.1 %). Furthermore, a higher proportion of patients with extrinsic-type lesions developed pelvic endometriosis, including endometrioma. Pelvic endometriosis is the progenitor of this type of adenomyosis. This finding points to the possibility that infiltration of the pelvic endometriosis from the uterine serosa to the myometrium may be involved in the onset of extrinsic adenomyosis. In fact, there are biological differences between the intrinsic and extrinsic lesion types [31]. The intramural type, which was not observed in any of the participants in our study, develops from Müllerian remnants, as opposed to being formed by infiltration of the endometrium or endometriotic tissues. The final type represents those lesions that do not belong to any of the abovementioned types; it is considered a heterogenous mixture of far advanced adenomyosis of the previous three types with an unknown mechanism of pathogenesis [15].

In this study, the groups were categorised using the Kishi classification. The presented trends, which showed high proportions of patients with a history of miscarriage in the intrinsic group and of patients with endometriosis in the extrinsic group, were similar to those reported by Kishi et al. [15]. However, the proportions among the classifications in our study differed greatly from those reported by Kishi et al. [15]. In their study, the proportion of the undetermined types (far advanced type) was 13.1 %, which was the lowest among the lesion types analysed; in our study, the proportion of the advanced group was the highest (76.9 %). No patients in our study had intramural-type lesions.

In the study by Kishi et al. [15], the patients underwent surgery for hypermenorrhoea or dysmenorrhoea, whereas our patients underwent IVF-ET for the chief complaint of infertility. Therefore, the differences in patients included in the two analyses may explain the discrepancies in the data. Particularly, women with advanced adenomyosis may have more difficulty attaining a live birth and may need IVF/ICSI-ET at higher rates than women with other types of adenomyosis.

Another possible reason for the differences in the data may be the method of patient selection. Among facilities that participated in the present joint study, none performed routine MRI for infertile patients. Patients do not undergo MRI unless deemed necessary by the attending gynaecologist, even when adenomyosis is diagnosed by transvaginal ultrasonography. Therefore, patients who were not diagnosed by transvaginal ultrasonography were not included in this study. Moreover, the patients with adenomyosis diagnosed by transvaginal ultrasonography whose gynaecologists did not order an MRI were similarly excluded. This may explain why patients with circumferential adenomyosis, which causes uterine enlargement, or advanced-type adenomyosis that involves full-thickness invasion with endometrial tissue, were overrepresented in our study. Considering that our cohorts were selected among infertile patients, the proportion of patients who developed endometriosis was high among the overall sample. This may similarly explain the higher proportion of patients with extrinsic-type than intrinsic-type lesions.

In this study, the rate of pregnancy loss was lower and the rate of live births was higher in the extrinsic group than in the advanced group. The rate of miscarriages at 12 weeks or later tended to be particularly high in the advanced group. Several previous studies have reported that the association between adenomyosis and infertility was stronger in patients with JZ or myometrium thickness, which are considered signs of adenomyosis progression [23, 32, 33]. Moreover, adenomyosis is associated with late miscarriage and preterm labour in patients with impaired deep placentation, which is defined by the presence of abnormalities of the JZ, involved in implantation, and absent or incomplete remodelling of the JZ segment of the spiral arteries [34, 35]. Patients with the extrinsic type were classified as those with adenomyotic lesions on the external side, which indicates that their JZ was comparatively better maintained than the JZ in patients with advanced- and intrinsic-type lesions. Overall, the extrinsic group (intact JZ) had fewer pregnancy losses and more live births. Furthermore, the advanced group was characterised by more miscarriages in late pregnancy, which may indicate an involvement of impaired and deep placentation.

Although the pathogenesis of advanced-type lesions remains unknown, many of our patients presumably had adenomyosis that progressed from the side of the uterine serosa, which is involved in uterine endometriosis, particularly given the high number of patients with endometrioma (70 %, 28/40) [15, 36]. Therefore, infertile patients with pelvic endometriosis should be assessed early for the presence and localisation of adenomyosis by MRI.

Late-term miscarriage, preeclampsia, placental malposition, and preterm delivery are some of the risks during pregnancy in patients with adenomyosis [17]. The rates of foetal growth restriction, intrauterine infection, and cervical incompetency similarly increase with adenomyosis [18]; these complications may possibly be due to environmental disorders, such as changes in cytokine production, caused by adenomyosis [9], or impaired deep placentation [35]. In this study, a large proportion of patients had threatened abortion or premature labour; 50 % required management for such conditions, even though they did not have premature labour.

There were several limitations to this study precluding its generalisability. First, this was a retrospective study; thus, only the available parameters could be included in the analysis. For example, concentrations of anti-Müllerian hormone, an indicator of ovarian reserve [37], could not be obtained. The IVF/ICSI fresh and frozen-thawed ET protocols were similarly determined by the respective gynaecologists at each facility; therefore, they were inconsistent. Furthermore, the transferred embryos included those in cleavage, morula, and blastocyst stages. The protocol used also involved pre-transfer GnRH treatment, which was observed to improve the outcomes of frozen ET in patients with adenomyosis [38]. In this study, this treatment was mostly performed in the advanced group; however, the extrinsic group had higher live birth and lower pregnancy loss rates than those noted in the advanced group. Although the efficacy of this treatment cannot be denied, it did not significantly affect the outcomes of the participants in this study. Second, because the study patients wanted to become pregnant, a histological diagnosis of adenomyosis was not possible. However, previous studies have reported that the diagnostic accuracy of MRI is adequate for adenomyosis; in this study, adenomyosis was diagnosed by two experienced radiologists. Thus, we believe that the accuracy of diagnosis was high. Third, the sample size was small, as it was limited to adenomyosis patients for whom MRI data were available. However, this allowed us to ensure adequate objectivity of adenomyosis classifications, as performed through an imaging assessment. In particular, because there were few patients with extrinsic- or intrinsic-type lesions, these two groups could not be compared. Although patients in these two groups had different characteristics, extrinsic and intrinsic adenomyotic lesions are believed to arise from different origins and are reported to have different pathologies [16]. Additional large-scale, prospective studies are warranted to assess how the types of adenomyosis affect fertility. Fourth, the population was heterogeneous due to the inclusion criteria. Since there were cases of adenomyosis derived from endometriosis, the confounding effect of endometriosis could not be excluded; furthermore, the existence of endometriosis could not be ruled out in cases in which laparotomy and laparoscopic surgery were not performed.

## Conclusions

Our findings suggest that adenomyotic lesions have different effects on IVF/ICSI fresh and frozen-thawed ET outcomes. One of the reasons why previous reports did not provide a consensus view on the effect of adenomyosis on ART outcomes is that they did not consider the localisation of adenomyotic lesions. Hence, future studies should aim to highlight the importance of MRI for treating infertile patients with suspected adenomyosis who complain of dysmenorrhoea or hypermenorrhoea. Establishing treatment plans according to adenomyotic lesion localisation should also be considered. We believe that our results will be useful for establishing such treatment plans.

## Supplementary Information

**Additional file 1**

## Data Availability

All data generated or analysed during this study are included in this article.
