# Supplementary material for: Effects of localisation of uterine adenomyosis on outcome of in vitro fertilisation/intracytoplasmic sperm injection fresh and frozen-thawed embryo transfer cycles: a multicentre retrospective cohort study
Source: Reprod Biol Endocrinol. 2021 Jun 4;19:84. doi: 10.1186/s12958-021-00764-7 (PMC8176737; doi:10.1186/s12958-021-00764-7)
Supplement: Supplementary file 1 — Additional file 1 [file 12958_2021_764_MOESM1_ESM.docx]

**Supplementary Information**

**History of disease and symptom of individual patients and their age**

**Advanced group**

|  | Age | Gravidity | Parity | Prior uterine surgery | Prior surgery for endometrioma | Hypermenorrhoea | Dysmenorrhoea |
| --- | --- | --- | --- | --- | --- | --- | --- |
| 1 | 36 | 0 | 0 | ＋ | ＋ | − | ＋ |
| 2 | 35 | 1 | 1 | ＋ | ＋ | ＋ | ＋ |
| 3 | 33 | 0 | 0 | − | − | ＋ | ＋ |
| 4 | 37 | 0 | 0 | ＋ | ＋ | − | ＋ |
| 5 | 36 | 0 | 0 | − | ＋ | ＋ | ＋ |
| 6 | 36 | 2 | 0 | − | − | ＋ | ＋ |
| 7 | 37 | 1 | 0 | − | − | ＋ | − |
| 8 | 39 | 1 | 1 | − | − | ＋ | ＋ |
| 9 | 37 | 0 | 0 | − | ＋ | − | ＋ |
| 10 | 32 | 1 | 0 | − | ＋ | ＋ | ＋ |
| 11 | 35 | 0 | 0 | − | − | ＋ | − |
| 12 | 37 | 0 | 0 | − | − | ＋ | ＋ |
| 13 | 33 | 0 | 0 | − | − | − | − |
| 14 | 41 | 0 | 0 | − | − | ＋ | ＋ |
| 15 | 37 | 2 | 1 | ＋ | − | ＋ | ＋ |
| 16 | 33 | 0 | 0 | − | − | ＋ | ＋ |
| 17 | 37 | 1 | 1 | − | ＋ | ＋ | ＋ |
| 18 | 28 | 0 | 0 | − | − | ＋ | ＋ |
| 19 | 33 | 0 | 0 | − | ＋ | − | ＋ |
| 20 | 41 | 2 | 0 | − | − | ＋ | ＋ |
| 21 | 36 | 1 | 0 | − | ＋ | ＋ | ＋ |
| 22 | 33 | 1 | 1 | − | − | ＋ | ＋ |
| 23 | 39 | 0 | 0 | − | − | ＋ | ＋ |
| 24 | 34 | 1 | 0 | − | ＋ | ＋ | ＋ |
| 25 | 36 | 1 | 0 | − | − | ＋ | ＋ |
| 26 | 35 | 0 | 0 | − | − | ＋ | ＋ |
| 27 | 37 | 0 | 0 | − | ＋ | ＋ | ＋ |
| 28 | 28 | 2 | 1 | − | − | − | − |
| 29 | 26 | 0 | 0 | − | − | ＋ | ＋ |
| 30 | 34 | 0 | 0 | − | − | − | − |
| 31 | 40 | 0 | 0 | − | − | − | ＋ |
| 32 | 40 | 0 | 0 | − | − | ＋ | ＋ |
| 33 | 37 | 1 | 0 | − | ＋ | − | ＋ |
| 34 | 35 | 0 | 0 | − | − | − | ＋ |
| 35 | 49 | 1 | 0 | − | ＋ | − | ＋ |
| 36 | 37 | 1 | 0 | − | − | ＋ | ＋ |
| 37 | 36 | 0 | 0 | − | − | ＋ | ＋ |
| 38 | 41 | 1 | 0 | − | − | ＋ | ＋ |
| 39 | 37 | 2 | 1 | − | − | ＋ | ＋ |
| 40 | 38 | 0 | 0 | ＋ | − | − | ＋ |

**Extrinsic group**

|  | Age | Gravidity | Parity | Prior uterine surgery | Prior surgery for endometrioma | Hypermenorrhoea | Dysmenorrhoea |
| --- | --- | --- | --- | --- | --- | --- | --- |
| 1 | 37 | 1 | 1 | ＋ | ＋ | − | ＋ |
| 2 | 37 | 0 | 0 | − | ＋ | ＋ | ＋ |
| 3 | 30 | 0 | 0 | ＋ | ＋ | ＋ | ＋ |
| 4 | 33 | 1 | 1 | − | − | ＋ | ＋ |
| 5 | 31 | 0 | 0 | − | ＋ | ＋ | ＋ |
| 6 | 37 | 1 | 1 | ＋ | ＋ | ＋ | ＋ |
| 7 | 33 | 1 | 0 | − | ＋ | − | − |
| 8 | 39 | 0 | 0 | − | ＋ | − | − |
| 9 | 37 | 0 | 0 | − | − | − | ＋ |

**Intrinsic group**

|  | Age | Gravidity | Parity | Prior uterine surgery | Prior surgery for endometrioma | Hypermenorrhoea | Dysmenorrhoea |
| --- | --- | --- | --- | --- | --- | --- | --- |
| 1 | 36 | 1 | 0 | − | − | ＋ | ＋ |
| 2 | 31 | 0 | 0 | − | ＋ | − | ＋ |
| 3 | 35 | 1 | 0 | − | − | − | − |
